# Potential Clinical Value of Pretreatment De Ritis Ratio as a Prognostic Biomarker for Renal Cell Carcinoma

**DOI:** 10.3389/fonc.2021.780906

**Published:** 2021-12-21

**Authors:** Jinze Li, Dehong Cao, Lei Peng, Chunyang Meng, Zhongyou Xia, Yunxiang Li, Qiang Wei

**Affiliations:** ^1^ Department of Urology, Institute of Urology, West China Hospital, Sichuan University, Chengdu, China; ^2^ West China School of Medicine, Sichuan University, Chengdu, China; ^3^ Department of Urology, Nanchong Central Hospital, The Second Clinical Medical College, North Sichuan Medical College, Nanchong, China

**Keywords:** De Ritis ratio, renal cell carcinoma, biomarker, prognosis, survival

## Abstract

**Background:**

We performed this study to explore the prognostic value of the pretreatment aspartate transaminase to alanine transaminase (De Ritis) ratio in patients with renal cell carcinoma (RCC).

**Methods:**

PubMed, EMBASE, Web of Science, and Cochrane Library were searched to identify all studies. The hazard ratio (HR) with a 95% confidence interval (CI) for overall survival (OS) and cancer-specific survival (CSS) were extracted to evaluate their correlation.

**Results:**

A total of 6,528 patients from 11 studies were included in the pooled analysis. Patients with a higher pretreatment De Ritis ratio had worse OS (HR = 1.41, p < 0.001) and CSS (HR = 1.59, p < 0.001). Subgroup analysis according to ethnicity, disease stage, cutoff value, and sample size revealed that the De Ritis ratio had a significant prognostic value for OS and CSS in all subgroups.

**Conclusions:**

The present study suggests that an elevated pretreatment De Ritis ratio is significantly correlated with worse survival in patients with RCC. The pretreatment De Ritis ratio may serve as a potential prognostic biomarker in patients with RCC, but further studies are warranted to support these results.

## Introduction

Renal cell carcinoma (RCC) is a common malignant tumor in adults, and its incidence has been increasing over the past two decades (1). In 2020, approximately 73,750 new RCC cases and 14,830 deaths were predicted in the United States (2). Despite an increase in early detection of RCC, nearly 20% of patients already have local progression or metastasis disease at initial diagnosis (3). Moreover, postoperative cancer recurrence occurs in 20%–40% of patients with localized RCC (4). Thus, it is of great value to define the prognostic indicators of survival, metastasis, or recurrence in patients with RCC.

Tumor, node, and metastasis (TNM) staging is an essential traditional prognostic factor for RCC, with limited accuracy when used alone (5, 6). Numerous clinical prognostic or predictive factors have been identified based on clinical trials and retrospective univariate or multivariate analysis, including performance status, appearing symptoms, and paraneoplastic syndromes (7–9). Besides, laboratory values were also used for prognosis, such as serum protein, corrected calcium, erythrocyte sedimentation rate, and neutrophil to lymphocyte ratio (10–12).

Aspartate transaminase (AST) and alanine transaminase (ALT) are the most critical transaminase in the body, reflecting hepatocellular damage (13). The ratio of serum AST to ALT, also known as the De Ritis ratio, is usually used to identify the etiology of various hepatitis (14). Recent studies have confirmed that the De Ritis ratio is a biomarker that can predict the prognosis of several tumors, such as breast cancer, gastric adenocarcinoma, and nasopharyngeal cancer (15–17). However, the prognostic value of this ratio in patients with RCC remains unclear. Bezan et al. (18) found that patients with a high De Ritis ratio had inferior overall survival (OS) and metastasis-free survival (MFS), while another study reported no correlation between high DR Ritis rate and OS (19). Therefore, this study aims to explore the prognostic value of the pretreatment De Ritis ratio in patients with RCC and provide higher-level medical evidence for clinical practice.

## Materials and Methods

### Search Strategy

This present study was performed following the Preferred Reporting Items for Systematic Reviews and Meta-Analyses (PRISMA) criteria (20) and was registered in PROSPERO (ID: CRD42021255149). PubMed, EMBASE, Web of Science, and Cochrane Library were searched to identify eligible studies up to April 2021 (update on October 28, 2021) without language restriction. The search items were as follows: renal cell carcinoma (renal cell cancer, renal carcinoma, kidney cancer, kidney neoplasms, clear cell carcinoma, adenocarcinoma, RCC), De Ritis ratio (aspartate transaminase, AST, alanine transaminase, ALT, aspartate transaminase/alanine transaminase ratio, AST/ALT ratio, AST to ALT ratio), and prognosis (recurrence, survival, outcome) as keywords or Mesh term. A list of references to relevant studies was also manually searched. Two authors reviewed the literature independently, and any differences settled through discussion with a third author.

### Inclusion and Exclusion Criteria

Qualified studies should meet the following inclusion criteria: (1) cohort studies or observational studies; (2) patients with RCC were histopathologically confirmed; (3) the pretreatment De Ritis ratio was obtained, (4) estimating the relationship between the De Ritis ratio and RCC prognosis; (5) reported available data for analysis, including OS or cancer-specific survival (CSS). Studies excluded were based on the following criteria: (1) studies involving animals; (2) reviews, comments, letters, case reports, and unpublished articles; (3) studies with unavailable data or insufficient data for analyses; (4) duplicated studies based on the same cohort.

### Data Extraction and Quality Assessment

Two reviewers independently extracted the required data from eligible studies, which were as follows: the first author’s name, year of publication, study region, study design, tumor type, treatment, sample size, patient age, the cutoff value of the De Ritis ratio, analysis method, and follow-up period. Furthermore, all outcome parameters were directly extracted with hazard ratio (HR) and 95% confidence interval (CI). The primary outcome was OS, while the secondary outcome was CSS. When both univariate and multivariate analyses were used in the study, data were extracted from the multivariate analysis. The quality of all included studies was estimated using the Newcastle–Ottawa scale (maximum score 9) (21). In the current study, we considered a study with a score of 7 or higher as a high-quality study (22). All discrepancies were discussed through negotiation or finally decided by a third reviewer.

### Statistical Analyses

The statistical analysis of this study was performed using Stata v.15.0 (Stata Corp, College Station, TX, USA). The merged HRs with 95% CIs were adopted to evaluate the correlation between the pretreatment De Ritis ratio and prognosis. Heterogeneity between studies was estimated using Cochran’s Q and I^2^ tests. p < 0.10 or I^2^ > 50% represented a significant heterogeneity. A random-effect model was applied for this meta-analysis. Moreover, we performed a subgroup analysis to investigate the cause of heterogeneity. Sensitivity analysis was also performed by dropping each study individually to assess the stability of the findings. Publication bias was assessed by using Begg’s test, as the small number of included studies. Statistical significance was defined as a p value of less than 0.05.

## Results

### Study Characteristics

**Figure 1:**
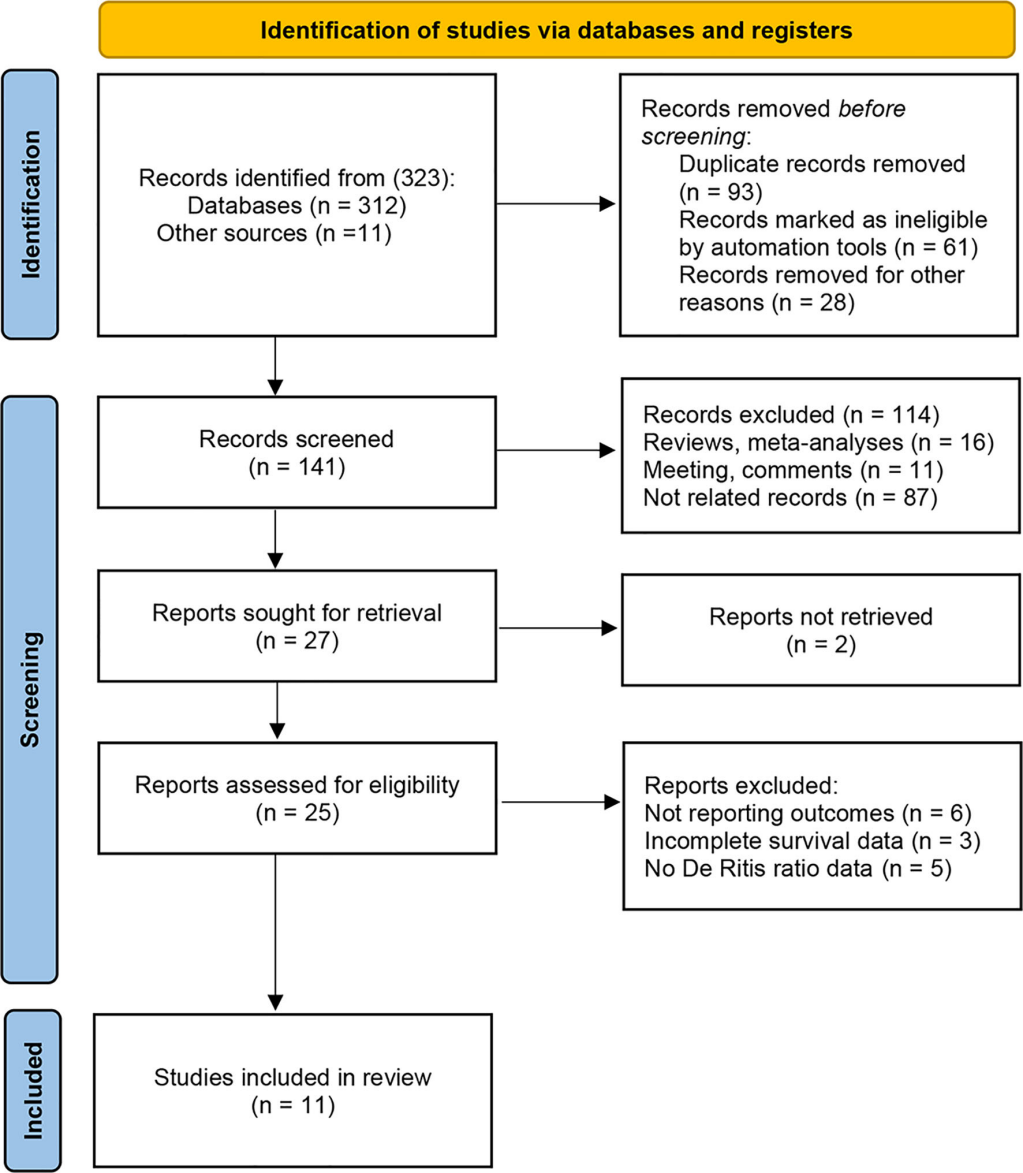
Flow diagram of studies identified, excluded, and included.

Of the 323 initially identified articles through the search strategy, 141 studies remained after removing duplicates (93 publications) and irrelevant studies (89 publications). Subsequently, 114 articles were excluded by viewing titles and abstracts (16 reviews or meta-analysis, 11 meetings or comments, and 87 not related records). Moreover, the full text of 2 articles could not be found. After full-text evaluation, 12 studies were excluded from the remaining 25 potential studies, including 6 without survival outcomes, 3 without adequate survival data, and 5 without De Ritis ratio data. Finally, eleven articles comprising 6,258 patients were included in the present analysis (18, 19, 23–31) (**Figure 1**). **Table 1** records the basic characteristics of all included studies. All studies had a retrospective design, two of which were propensity score-matched analyses. Five studies focused on metastatic RCC (24, 26, 27, 30, 31). Six studies focused on non-metastatic RCC (18, 19, 23, 25, 28, 29). These studies were conducted in many countries, including China, Korea, Turkey, Japan, Germany, the United States, and European countries. The median age of patients included in the study ranged from 55 to 65 years. The cutoff values for the De Ritis ratio ranged from 1.0 to 1.5. The median follow-up period for the included studies ranged from 21 to 60 months, and only one study did not report the follow-up period (27). Ten studies recorded the association between De Ritis ratio and OS, and seven studies recorded CSS. All studies were regarded as high-quality based on the NOS score, and the specific quality score of each study is shown in **Supplementary File 1**.

**Table 1:** Baseline characteristics of include studies and methodological assessment.

Authors (year)	Region	Study design	Tumor type	Treatment	Number of patients	Age (years)	Cutoff value (AST/ALT)	Analysis method	Outcomes	Follow-up (months)	Quality score
Bezan 2015 (18)	America	Retrospective	Non-metastatic	Surgery	698	Median 65.4 (55.8–73.4)	1.26	Multivariate	OS	Median 60	8
Canat 2017 (19)	Turkey	Retrospective	Non-metastatic	Surgery	298	Median 61 (22–86)	1.5	Univariate	OS, CSS	Mean 37.8 ± 22.3	7
Gu 2017 (23)	China	Retrospective	Non-metastatic	Surgery	185	Mean 56.1 ± 11.8	1.0	Univariate	OS	Median 30.2 (12.1–48.4)	8
Ishihara 2017 (24)	Japan	Propensity score matching	Metastatic	Surgery	118	Median 65	1.24	Multivariate	OS, CSS	Mean 21.0 ± 24.3	9
Lee 2017 (25)	Korea	Propensity score matching	Non-metastatic	Surgery	2965	Median 55 (47–65)	1.5	Multivariate	OS, CSS	Median 37 (24–73)	9
Kang 2018 (26)	Korea	Retrospective	Metastatic	TKI	360	Median 58 (51–67)	1.2	Multivariate	OS, CSS	Median 29 (24.1–33.9)	9
Kim 2018 (27)	Korea	Retrospective	Metastatic	TT	158	Mean 58.6 ± 10.6	1.38	Univariate	OS,	NR	7
Ikeda 2020 (28)	Japan	Retrospective	Non-metastatic	Surgery	243	Median 61 (55–67)	1.42	Multivariate	CSS	Median 60 (25–103)	9
Kang 2020 (29)	Korea	Retrospective	Non-metastatic	Surgery	670	Median 55 (48–61)	1.0	Univariate	OS, CSS	Median 59 (41–81)	8
Laukhtina 2020 (30)	Europe and America	Retrospective	Metastatic	Surgery	613	Median 57 (50–64)	1.2	Multivariate	OS, CSS	Median 31 (16–58)	9
Janisch 2021 (31)	Germany	Retrospective	Metastatic	TKI	220	Median 64 (57–71)	1.08	Multivariate	OS	Median 28 (10–58)	9

TKI, tyrosine kinase inhibitor; TT, targeted therapy; AST, aspartate transaminase; ALT, alanine transaminase; OS, overall survival.

CSS, cancer-specific survival; PFS, progression-free survival; MFS, metastasis-free survival; NR, not report.

### Overall Survival

**Figure 2:**
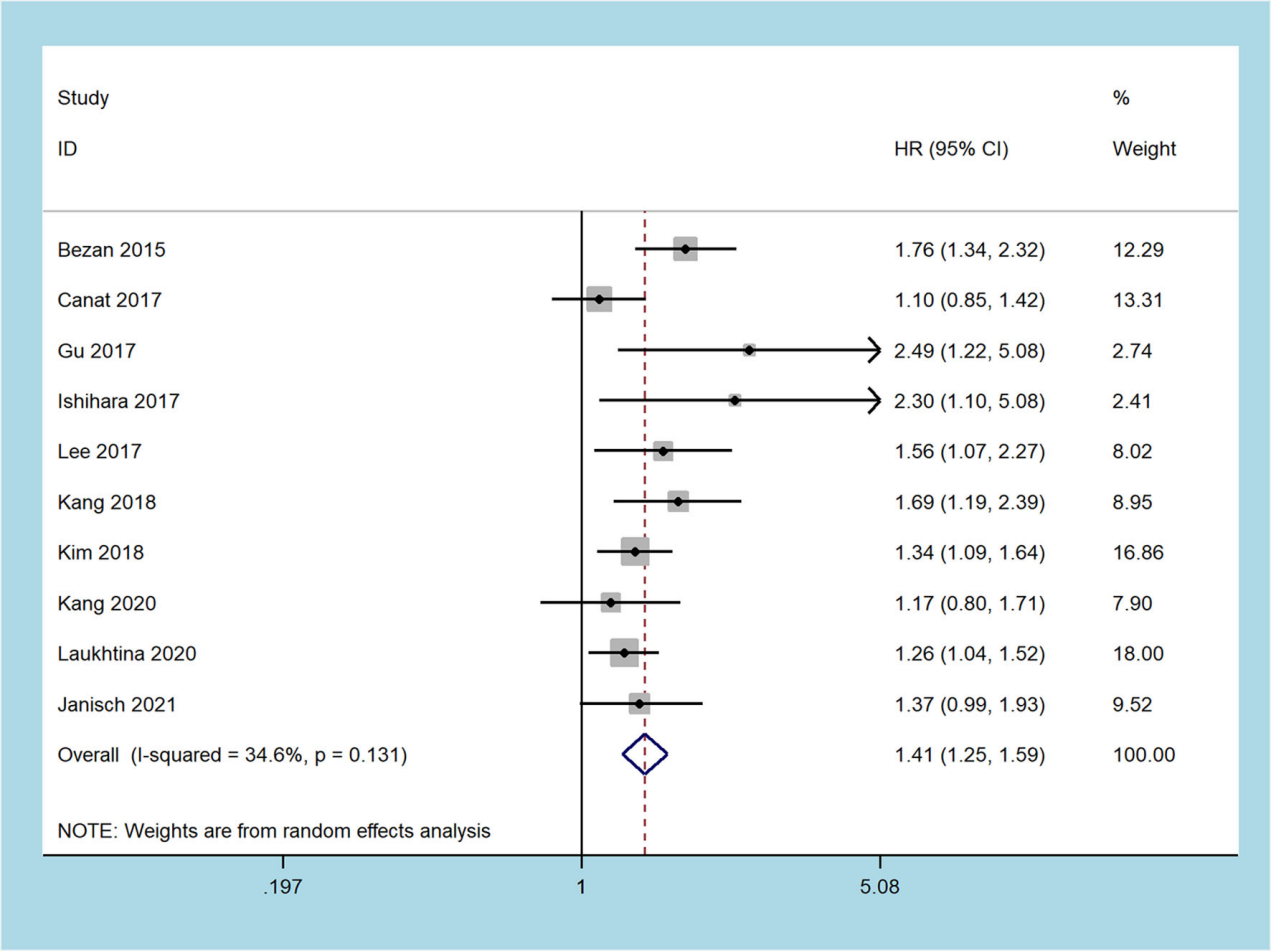
Forest plots of the association between the De Ritis ratio and overall survival.

Nine studies including 6,285 patients recorded about OS (18, 19, 23–27, 29–31). Since moderate heterogeneity was found, the random-effect model was adopted (I2 = 34.6%, p = 0.131). The merged results demonstrated that patients with an increased pretreatment De Ritis ratio had inferior OS (HR = 1.41, 95% CI 1.25 to 1.59, p < 0.001, **Figure 2**).

### Cancer-Specific Survival

**Figure 3:**
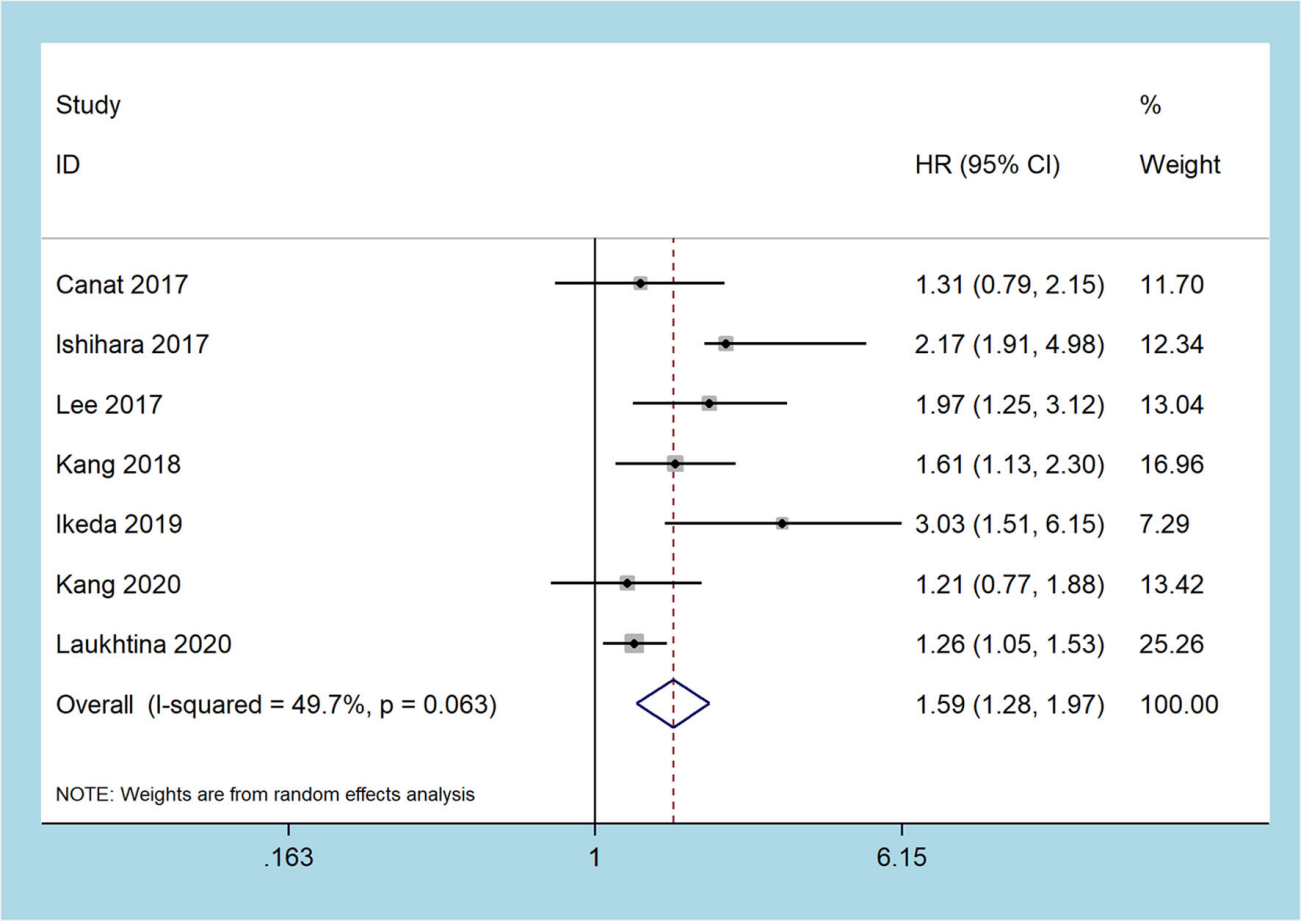
Forest plots of the association between the De Ritis ratio and cancer-specific survival.

Seven studies recorded the prognostic role of the pretreatment De Ritis ratio in patients with RCC on CSS, including 5,167 patients (19, 24–26, 28–30). The pooled results revealed that a higher pretreatment De Ritis ratio was related to worse CSS (random-effect model: HR = 1.59, 95% CI 1.28 to 1.97, p < 0.001), and with moderate heterogeneity (I2 = 49.7%, p = 0.063, **Figure 3**).

### Subgroup Analyses

Limited to the number of studies included in the meta-analysis, we only conducted subgroup analysis for OS and CSS oncologic outcomes, and stratified by ethnicity, disease stage, treatment method, cutoff value, analysis method, or sample size (**Table 2**). For studies that include the Asian population, the higher pretreatment De Ritis ratio was associated with inferior OS (HR = 1.49, 95% CI 1.25 to 1.77, p < 0.001, I^2^ = 20.5%) and CSS (HR = 1.80, 95% CI 1.38 to 2.33, p < 0.001, I^2^ = 35.6%). Moreover, in the Caucasian population subgroup, the high De Ritis ratio was also an independent predictor of OS (HR = 1.34, 95% CI 1.11 to 1.62, p = 0.002, I^2^ = 53.4%) and CSS (HR = 1.27, 95% CI 1.06 to 1.51, p = 0.009, I^2^ = 0%). Subgroup analysis by disease stage demonstrated that the high pretreatment De Ritis ratio was related to worse OS (HR = 1.37, 95% CI 1.21 to 1.54, p < 0.001, I^2^ = 0%) and CSS (HR = 1.54, 95% CI 1.14 to 2.08, p < 0.001, I^2^ = 60.1%) in patients with metastatic RCC, and similar results were observed in patients with non-metastatic RCC (OS: HR = 1.45, 95% CI 1.13 to 1.86, p = 0.004, I^2^ = 58.9%; CSS: HR = 1.66, 95% CI 1.15 to 2.40, p < 0.001, I^2^ = 50.7%). In terms of subgroup analysis for the treatment method, the high pretreatment De Ritis ratio in patients with RCC was an independent predictor of OS (surgery: HR = 1.43 95% CI 1.18 to 1.72, p < 0.001, I^2^ = 51.3%; non-surgery: HR = 1.41, 95% CI 1.25 to 1.72, p < 0.001, I^2^ = 0%). For the subgroup with a cutoff value of > 1.2, the patients with a higher pretreatment De Ritis ratio had poor OS (HR = 1.44, 95% CI 1.18 to 1.76, p < 0.001, I^2^ = 51.1%) and CSS (HR = 1.94, 95% CI 1.43 to 2.6, p < 0.001, I^2^ = 27.3%). Likewise, in the cutoff value of the ≤1.2 group, the increased De Ritis ratio was correlated with worse OS (HR = 1.39, 95% CI 1.17 to 1.65, p < 0.001, I^2^ = 27.0%) and CSS outcomes (HR = 1.45, 95% CI 1.15 to 1.83, p < 0.001, I^2^ = 0%). In the multivariate analysis subgroup, the high pretreatment De Ritis ratio was related to poor OS (HR = 1.50, 95% CI 1.29 to 1.58, p < 0.001, I^2^ = 21.7%) and CSS (HR = 1.76, 95% CI 1.32 to 2.35, p < 0.001, I^2^ = 63.5%). In the univariate analysis subgroup, a higher pretreatment De Ritis ratio had worse OS (HR = 1.28, 95% CI 1.04 to 1.58, p = 0.020, I^2^ = 40.8%) but not in CSS (HR = 1.25, 95% CI 0.90 to 1.75, p = 0.184, I^2^ = 0.0%). Additionally, stratified by sample size, the higher pretreatment De Ritis ratio had steep inferior OS (HR = 1.45, 95% CI 1.23 to 1.71, p < 0.001, I^2^ = 34.1%) and CSS (HR = 1.42, 95% CI 1.28 to 1.73, p = 0.001, I^2^ = 29.8%) in the sample size >300 subgroup, which was consistent with the results of the sample size ≤300 subgroup (OS: HR = 1.38, 95% CI 1.12 to 1.71, p = 0.003, I^2^ = 43.3%; CSS: HR = 1.97, 95% CI 1.25 to 3.10, p = 0.004, I^2^ = 51.4%).

**Table 2:** Subgroup analyses of OS and CSS.

Outcome	Variable	No. of studies	Model	HR (95% CI)	p	Heterogeneity		Pm
						I^2^ (%)	p	
**OS**	All	10	Random	1.41 (1.25, 1.59)	< 0.001	34.6	0.131	
Ethnicity	Asian Caucasian	6 4	Random Random	1.49 (1.25, 1.77) 1.34 (1.11, 1.62)	< 0.001 0.002	20.5 53.4	0.279 0.092	0.409
Disease stage	Metastatic Non-metastatic	5 5	Random Random	1.37 (1.21, 1.54) 1.45 (1.13, 1.86)	< 0.001 0.004	0.0 58.9	0.414 0.045	0.935
Treatment method	Surgery Non-surgery	7 3	Random Random	1.43 (1.18, 1.72) 1.41 (1.25, 1.65)	< 0.001 < 0.001	51.3 0.0	0.055 0.521	0.897
Cutoff value	>1.2 ≤1.2	5 5	Random Random	1.44 (1.18, 1.76) 1.39 (1.17, 1.65)	<0.001 <0.001	51.1 27.0	0.085 0.242	0.877
Analysis method	Multivariate Univariate	6 4	Random Random	1.50 (1.29, 1.73) 1.28 (1.04, 1.58)	<0.001 0.020	21.7 40.8	0.271 0.167	0.220
Sample size	>300 ≤300	5 5	Random Random	1.45 (1.23, 1.71) 1.38 (1.12, 1.71)	<0.001 0.003	34.1 43.3	0.194 0.132	0.629
**CSS**	All	7	Random	1.59 (1.28, 1.97)	<0.001	49.7	0.063	
Ethnicity	Asian Caucasian	5 2	Random Random	1.80 (1.38, 2.33) 1.27 (1.06, 1.51)	<0.001 0.009	35.6 0.0	0.184 0.887	0.086
Disease stage	Metastatic Non-metastatic	3 4	Random Random	1.54 (1.14, 2.08) 1.66 (1.15, 2.40)	<0.001 <0.001	60.1 50.7	0.082 0.107	0.809
Cutoff value	>1.2 ≤1.2	4 3	Random Random	1.94 (1.43, 2.64) 1.45 (1.15, 1.83)	<0.001 0.001	27.3 0.0	0.248 0.063	0.067
Analysis method	Multivariate Univariate	5 2	Random Random	1.76 (1.32, 2.35) 1.25 (0.90, 1.75)	<0.001 0.184	63.5 0.0	0.027 0.816	0.278
Sample size	>300 ≤300	4 3	Random Random	1.42 (1.28, 1.73) 1.97 (1.25, 3.10)	0.001 0.004	29.8 51.4	0.233 0.128	0.250

OS, overall survival; CSS, cancer-specific survival; HR, hazard ratio; CI, confidence interval.

### Sensitivity Analysis and Meta-Regression Analysis

Restricted to the number of articles included in the study, we performed a sensitivity analysis for OS and CSS outcomes. After performing the leave-one-out test or excluding small studies (<200 patients), no significant change in the pooled HR was observed, which undoubtedly proved the reliability of our results (**Supplementary File 2**). We also performed a meta-regression analysis to explore the suspected reasons for the heterogeneity of OS and CSS outcomes. The results showed that ethnicity (p = 0.409), disease stage (p = 0.935), treatment method (p = 0.897), cutoff value (p = 0.877), analysis method (p = 0.220), and sample size (p = 0.692) did not significantly affect the heterogeneity of OS. In addition, ethnicity (p = 0.086), disease stage (p = 0.809), cutoff value (p = 0.067), analysis method (p = 0.278), and sample size (p = 0.250) had no influence on CSS heterogeneity (**Table 2**).

### Publication Bias

**Figure 4:**
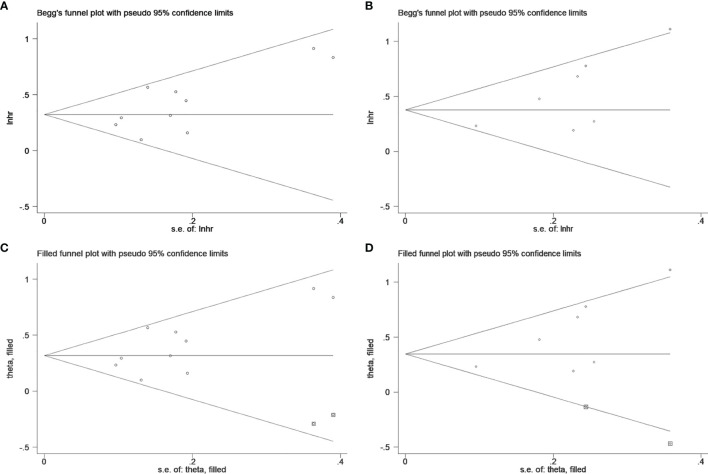
Begg’s test for **(A)** overall survival and **(B)** cancer-specific survival; Trim and fill method for **(C)** overall survival and **(D)** cancer-specific survival.

Begg’s test was applied to estimate the publication bias. A visual inspection of Begg’s funnel plots revealed asymmetry (**Figure 4**). This raises the possibility of publication bias, although the Begg’s test was not statistically significant (OS: p = 0.152, CSS: p = 0.072). Because of this, we used the trim and fill method to further detect publication bias, and two filled funnel plots demonstrated that even if the uncollected literature was included, it did not affect the results of the combined effect, which indicates that our results are relatively robust (**Figure 4**).

## Discussion

RCC is one of the most common solid lesions in the kidney, accounting for about 80%–90% of all renal malignancies (1). The prognosis of RCC is affected by various factors, including patient age, clinical manifestations, laboratory values, and tumor pathologic variables such as pathological stage, nuclear grade, and histological subtype (32, 33). Tumor stage and grade are considered as common prognostic markers for RCC, but the application of these factors in clinical practice remains problematic (34). How to more accurately identify those patients with poor prognosis before treatment and carry out the risk stratification of tumors are of great significance for choosing treatment options and the guidance of postoperative follow-up. Therefore, finding potential prognostic markers for RCC prognosis has become a hot spot in clinical research.

Initially, the serum De Ritis ratio was adopted to evaluate the prognosis of various liver diseases, including viral hepatitis, alcoholic hepatitis, and fatty liver (14). Because laboratory tests are routinely performed before treating cancer patients, the De Ritis ratio can be a simple, convenient, and inexpensive measurement method. Previous studies have reported that the De Ritis ratio was significantly associated with the prognosis of several tumors, including RCC (15–18). However, the actual prognostic value of this ratio in patients with RCC remains controversial. Su et al. (35) conducted a meta-analysis to explore the prognostic value of the De Ritis ratio in urological cancers, and 6 articles focused on RCC were included. They claimed that the patients with a higher De Ritis ratio had inferior OS (4 studies involved). A problem of the study by Su is their interpretation of OS since the data provided by the original research in their studies all indicated that an elevated De Ritis ratio had poor survival. Furthermore, a recent study reported that the De Ritis ratio was not associated with RCC prognosis (31). Thus, it is necessary to reevaluate the role of the De Ritis ratio in the prognosis of RCC based on the existing literature to better guide clinical practice.

Compared with the study by Su, the advantage of the current meta-analysis is that we included five more recent articles and eventually included 6,528 RCC patients for the analysis. The study revealed that patients with a higher pretreatment De Ritis ratio had worse survival outcomes regarding OS and CSS. Subgroup analyses of OS and CSS by ethnicity, disease stage, treatment method, cutoff value, analysis method, or sample size obtained similar results. Previous studies have suggested that histological subtypes are also prognostic factors for RCC (33). However, it was not possible to perform a subgroup analysis to assess the impact of different histological subtypes of the De Ritis ratio on prognosis due to lack of data. Remarkably, Lee et al. (25) found that a higher De Ritis ratio was associated with OS and CSS in localized clear-cell RCC patients, but not in non-clear-cell RCC. Janisch et al. (31) also revealed that an elevated De Ritis ratio was an unfavorable factor for OS in patients with clear-cell histology. Since these favorable results were obtained based on limited studies and insufficient sample sizes, therefore, a prospective large-scale cohort is needed to validate the conclusion.

It should be noted that sensitivity analyses indicated that our results were robust, but moderate heterogeneity among the included studies was found in both survival outcomes, a finding that may be due to different baseline characteristics of individual studies. Therefore, the meta-regression analysis was performed using ethnicity, disease stage, treatment method, cutoff value, analysis method, and sample size to explore the potential sources of heterogeneity. However, none of these factors can explain the heterogeneity of OS. Similar results were obtained for CSS. We also performed a subgroup analysis to investigate the cause of heterogeneity. The results showed that the heterogeneity of most subgroup analyses was slightly reduced, but it was still statistically significant in several subgroups. Thus, the random-effect model was used to calculate the effect size to minimize the impact of heterogeneity on the combined results. In addition, although we have conducted an extensive literature search, we still found a potential publication bias in the study reporting survival outcomes. We used the trim and fill method for further analysis and found that the inclusion of uncollected studies did not influence the results of the pooled effect, which suggests that publication bias may not have a significant effect on the overall findings. Therefore, the results of this study are relatively robust and reliable.

ALT and AST are often used to reflect hepatocellular damage or death. ALT is mainly present in the liver, while AST is widely distributed in various tissues such as the heart, liver, brain, muscle, and kidney tissues (14). Hence, ALT suggests liver disease specifically, while AST may be associated with several diseases that affect other organs. Pathological processes that have been proved to cause tissue damage, high proliferative states, and faster tumor cell turnover tend to enhance the serum AST level rather than ALT level, making the De Ritis (AST/ALT) ratio an attractive potential clinical biomarker (36).

Although the De Ritis ratio is a promising marker, the specific mechanism of this higher ratio and the inferior prognosis of cancer patients remain unclear. Indeed, cancer cells have a higher rate of glycolysis compared with normal cells, even in the presence of oxygen, and abnormal glycolytic metabolism produces sufficient ATP to promote cancer cell proliferation; this phenomenon is known as the “Warburg effect” (37, 38). Increased glycolysis in tumor cells is thought to be related to changes in nicotinamide adenine dinucleotide (NAD)-related enzymes and glucose transporters within mitochondria, according to Dorward et al. (39). A higher lactate dehydrogenase and cytosolic (NADH)/NAD+ ratio plays an essential role in maintaining enhanced glycolysis (40). It must be highlighted that AST is a pivotal component of the malate–aspartate shuttle in the glycolysis pathway that relocates NADH into mitochondria (14). Moreover, the previous study had confirmed that von Hippel-Lindau (VHL) significantly associated with renal clear-cell type RCC was presented in the cytoplasm of mitochondria (41). The loss of VHL and an increase in hypoxia-inducible factor expression influence several metabolic pathways, including glycolysis and oxidative phosphorylation (42). Accordingly, AST may be related to the glycolysis mechanism of clear-cell type RCC with VHL loss (25). However, further investigation is needed to explore the exact mechanism.

Considering that serum ALT and AST are commonly used indicators of clinical hematology, they are simple and easy to measure, and the cost is low. Therefore, the pretreatment De Ritis ratio can be used as an effective prognostic marker in patients with RCC and applied in clinical diagnosis and treatment. Our meta-analysis affirms that patients with an increased pretreatment De Ritis ratio had worse survival outcomes. It could be a potential selection criterion for the hierarchical management of risk factors for RCC (18). Given that a prognostic factor must be verified in well-designed, large-scale with an independent cohort before it can be applied universally, the findings should be interpreted cautiously.

Although the study provides more substantial evidence for the prognostic value of the pretreatment De Ritis ratio in patients with RCC, there are certain limitations. Firstly, the sample size of some of the included studies is relatively small, which may lead to a biased conclusion. Secondly, all included studies were retrospective, which may have an inherent structural bias, and the duration of follow-up was relatively short. Thirdly, similar to the study by Su (35), since this study only includes published literature, it may have potential publication bias. Fourth, patients could not be stratified according to histology due to lack of data. However, we conducted subgroup analysis based on sample size, study population, and disease status to explore potential sources of heterogeneity, which made our results more robust. Fifth, although the included studies attempted to exclude all patients with liver disease, there were still undetected liver pathological conditions that could affect the serum AST or ALT levels and distort the De Ritis ratio.

## Conclusion

Available evidence suggests that patients with an increased pretreatment De Ritis ratio have worse OS and CSS, indicating that this ratio may serve as a potential prognostic biomarker in RCC patients. However, prospective, well-designed, and large-scale studies are warranted to validate our findings.

## Data Availability Statement

The original contributions presented in the study are included in the article/**Supplementary Material**. Further inquiries can be directed to the corresponding authors.

## Ethics Statement

Ethical review and approval were not required for the study on human participants in accordance with the local legislation and institutional requirements. Written informed consent for participation was not required for this study in accordance with the national legislation and the institutional requirements.

## Author Contributions

YL and QW: conception and design. JL, DC, CM, and LP: acquisition of data and critical revision of the manuscript for important intellectual content. JL, DC, and ZX: analysis and interpretation of data. JL and DC: drafting of the manuscript. QW: supervision. All authors contributed to the article and approved the submitted version.

## Funding

This work was funded by the National Natural Science Foundation of China (Grant Number 82000721), Post-Doctor Research Project, West China Hospital, Sichuan University (Grant Number 2019HXBH089), Health Commission of Sichuan province (Grant Number 20PJ036), and the Sichuan Province Science and Technology Planning Project (Grant Numbers 2020YJ0054, 2020YFS0320).

## Conflict of Interest

The authors declare that the research was conducted in the absence of any commercial or financial relationships that could be construed as a potential conflict of interest.

## Publisher’s Note

All claims expressed in this article are solely those of the authors and do not necessarily represent those of their affiliated organizations, or those of the publisher, the editors and the reviewers. Any product that may be evaluated in this article, or claim that may be made by its manufacturer, is not guaranteed or endorsed by the publisher.
