# Supplementary material for: Potential Clinical Value of Pretreatment De Ritis Ratio as a Prognostic Biomarker for Renal Cell Carcinoma
Source: Front Oncol. 2021 Dec 21;11:780906. doi: 10.3389/fonc.2021.780906 (PMC8724044; doi:10.3389/fonc.2021.780906)

**Supplementary file 1.** Sensitivity analysis

**Figure S1.** Sensitivity analysis for overall survival (leave-one-out test).


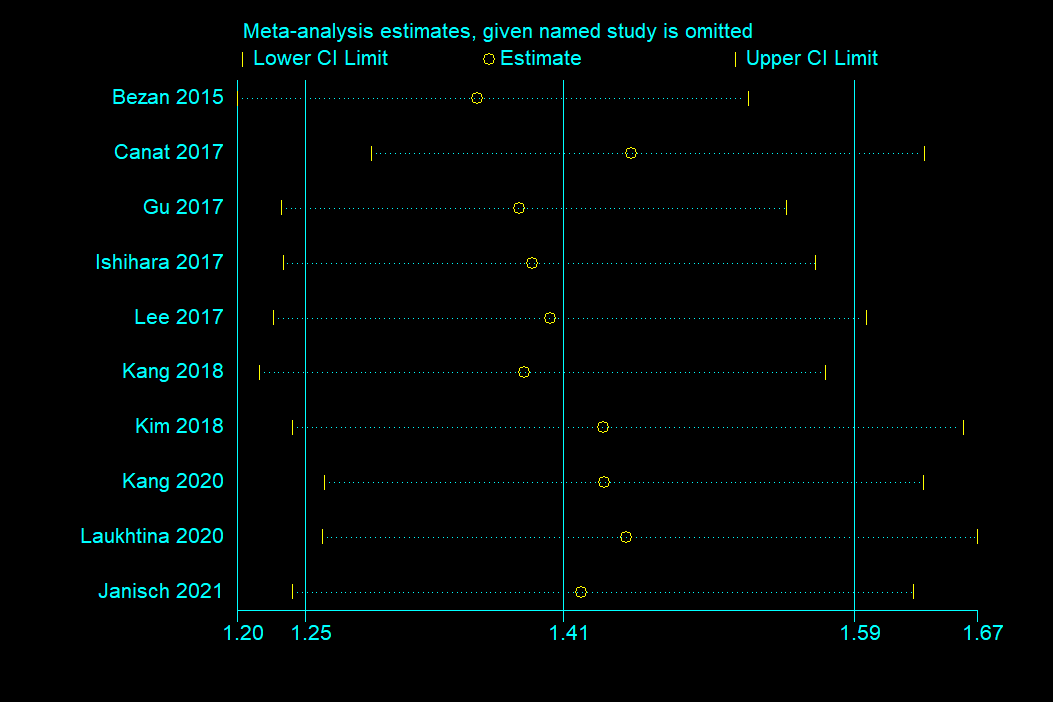


**Figure S2.** Sensitivity analysis for cancer-specific survival.


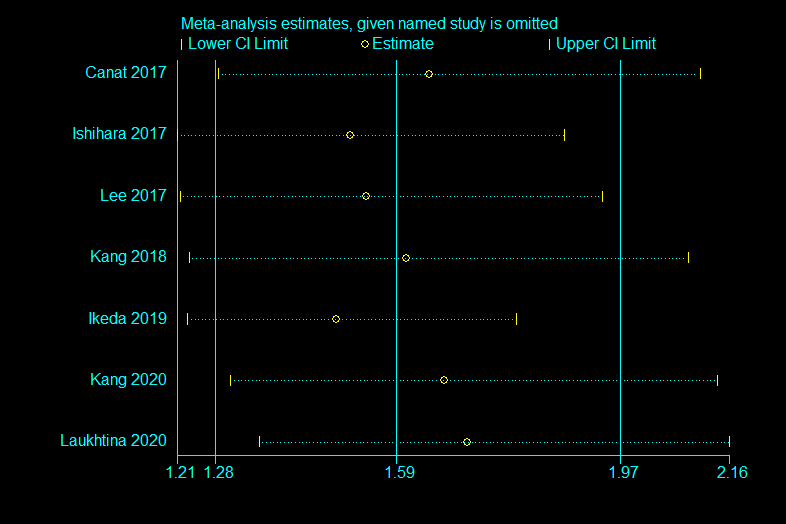


**Figure S3.** Forest plots of the association between De Ritis ratio and overall survival (excluding studies with < 200 patients).


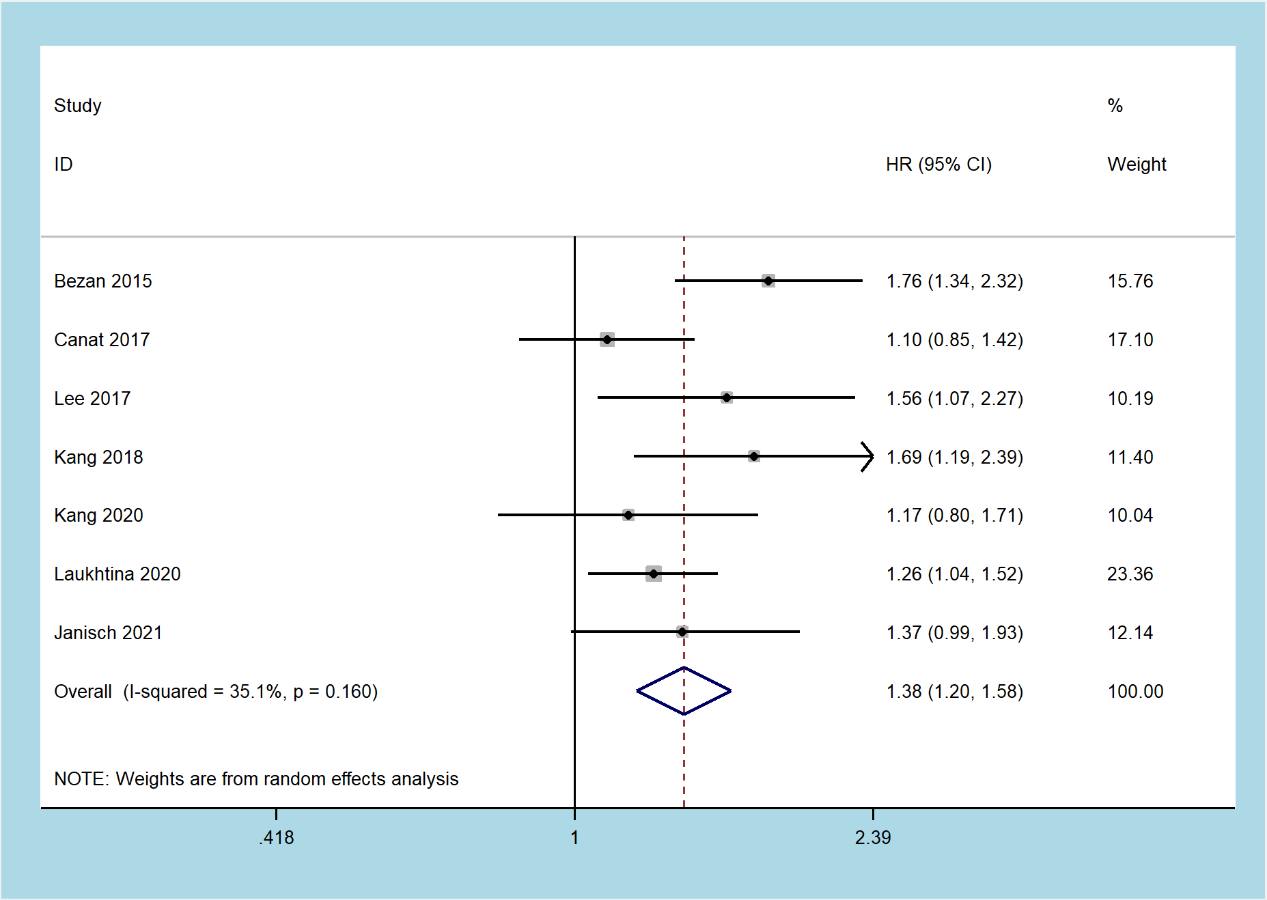


**Figure S4.** Forest plots of the association between De Ritis ratio and cancer-specific survival (excluding studies with < 200 patients).


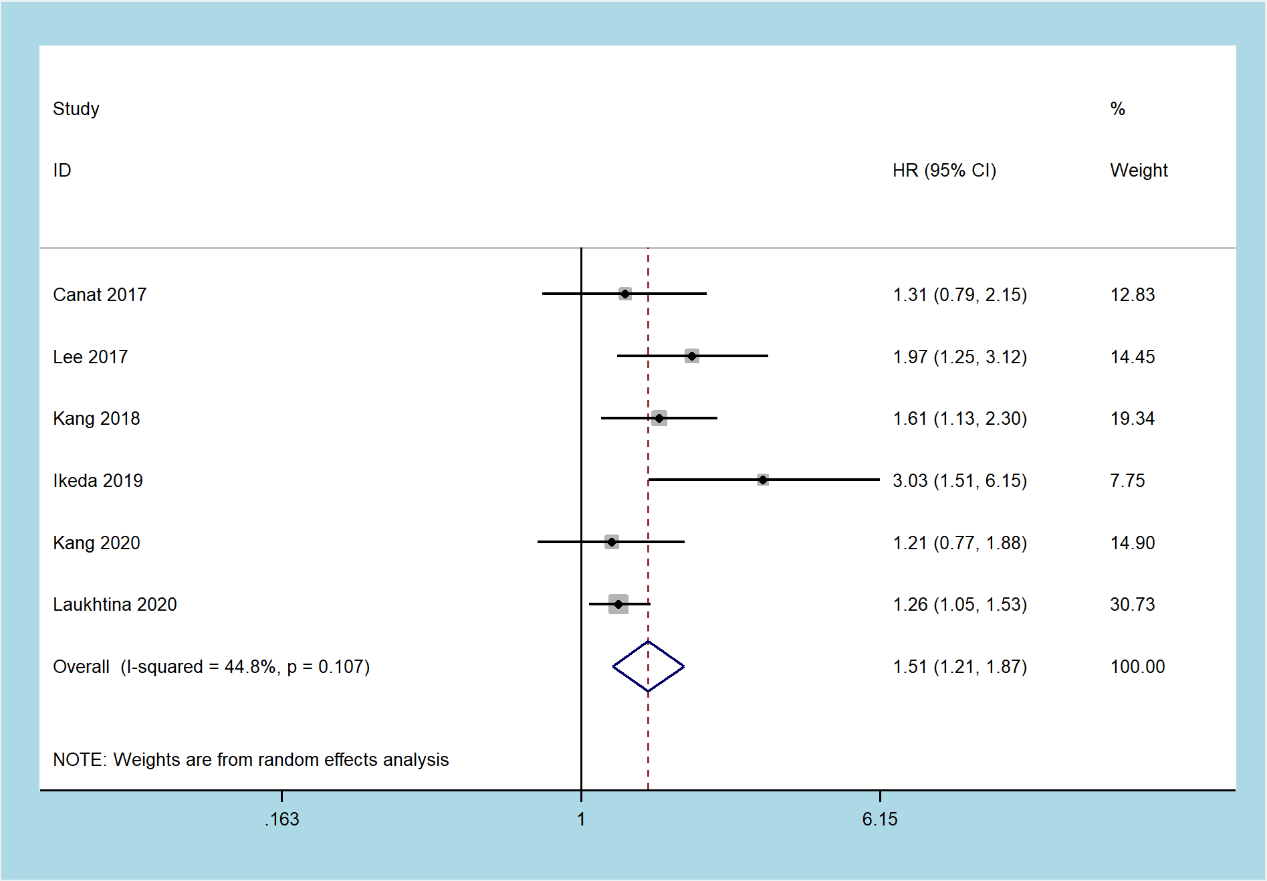

Supplement: Supplementary file 1 [file DataSheet_1.docx]
