# Supplementary material for: Potential Clinical Value of Pretreatment De Ritis Ratio as a Prognostic Biomarker for Renal Cell Carcinoma
Source: Front Oncol. 2021 Dec 21;11:780906. doi: 10.3389/fonc.2021.780906 (PMC8724044; doi:10.3389/fonc.2021.780906)
Supplement: Supplementary file 2 [file Table_1.docx]

| **Supplementary file 1.** Quality evaluation of the eligible studies with Newcastle–Ottawa scale. | | | | | | | | | |
| --- | --- | --- | --- | --- | --- | --- | --- | --- | --- |
| Study | Selection | | | | Comparability | | Outcome | | |
|  | Representativeness | Selection of  non-exposed | Ascertainment  of exposure | Outcome not present at start | Comparability on most important factors | Comparability on other risk factors | Assessment of outcome | Long enough follow-up (median≥1 year) | Adequacy  (completeness) of follow-up |
| Bezan 2015 | * | * | * | * | * | - | * | * | * |
| Canat 2017 | * | * | * | * | - | - | * | * | * |
| Gu 2017 | * | * | * | * | * | - | * | * | * |
| Ishihara 2017 | * | * | * | * | * | * | * | * | * |
| Lee 2017 | * | * | * | * | * | * | * | * | * |
| Kang 2018 | * | * | * | * | * | * | * | * | * |
| Kim 2018 | * | * | * | * | * | - | * | - | * |
| Ikeda 2020 | * | * | * | * | * | * | * | * | * |
| Kang 2020 | * | * | * | * | * | - | * | * | * |
| Laukhtina 2020 | * | * | * | * | * | * | * | * | * |
| Janisch 2021 | * | * | * | * | * | * | * | * | * |
| *indicates criterion met; - indicates significant of criterion not met. | | | | | | | | | |
